# Mapping evolutionary paradigm of Oropouche virus driven by dinucleotide bias and context-dependent codon bias

**DOI:** 10.1080/21505594.2025.2600128

**Published:** 2025-12-04

**Authors:** Xuanye Yang, Zeyu Liu, Xiaoting Ren, Chunlin Wang, Jianhua Zhou

**Affiliations:** a Key Laboratory of Biotechnology and Bioengineering of State Ethnic Affairs Commission, Biomedical Research Center, Northwest Minzu University, Lanzhou, China; b College of Life Science and Engineering, Northwest Minzu University, Lanzhou, Gansu, China

**Keywords:** Oropouche virus, evolutionary paradigm, nucleotide pair, synonymous codon, context-dependent codon bias

## Abstract

Arthropod-borne viruses (arboviruses) pose significant public health threats, and understanding their evolutionary mechanisms can inform molecular diagnostics and vaccine design. Oropouche virus (OROV), a relatively understudied arbovirus, exhibits unique evolutionary dynamics in nucleotide composition. We analyzed 45 OROV strains across genotypes to assess selective pressures shaping nucleoprotein, glycoprotein, and RNA-dependent RNA polymerase (RdRp) evolution. We utilized information entropy, dinucleotide odds ratios, relative synonymous codon usage values, and context-dependent codon bias (CDCB) to elucidate the genetic characteristics associated with mono-, di-, tri-, and tetranucleotide compositions. The extent of overall nucleotide usage bias, dinucleotide bias, and synonymous codon usage bias did not correlate with genotype-specific patterns, but rather exhibited a protein function-dependent pattern across the three proteins. Although dinucleotide bias and synonymous codon usage bias varied within a relatively broad range, the dinucleotide CpG, the over- and under-represented synonymous codons, and CDCB remained strongly influenced by natural selective pressures from both the host and the viral life cycle. Furthermore, the codon usage patterns, as indicated by the effective number of codons, suggest that the OROV nucleoprotein has been subject to stronger selective pressures in its evolutionary paradigm compared to the glycoprotein and RdRp, which appear to be primarily influenced by natural selection and mutation pressure. Additionally, analysis of relative codon deoptimization index (RCDI) and tRNA adaptation index (tAI) revealed suboptimal translational efficiency of OROV coding sequences in human hosts, suggesting limited codon usage adaptation.

## Introduction

According to the International Committee on Taxonomy of Viruses (ICTV) 2023–2024 release, Oropouche virus (OROV), which is a significant arbovirus, is taxonomically classified within the species *Orthobunyavirus oropouchense*, genus *Orthobunyavirus*, family *Peribunyaviridae*, order *Bunyavirales*. The OROV was initially endemic to the Amazon region, with reported cases in Bolivia, Brazil, Colombia, Ecuador, French Guiana, Panama, and Peru. Transmission to humans primarily occurs through bites from infected biting midges, although some mosquito species may also act as vectors. In late 2023, OROV was identified as the etiological agent responsible for significant outbreaks in previously endemic areas of the Amazon, raising concerns about its potential role in future emerging epidemics both regionally and globally [1–4]. OROV infection typically presents as an acute febrile illness characterized by sudden onset of high fever, severe headache, chills, myalgia, and arthralgia. This clinical profile frequently leads to misdiagnosis as other arboviral diseases, such as dengue, chikungunya, and Zika viruses, as well as malaria. Currently, no licensed vaccines or specific antiviral therapies exist for OROV. Prevention relies exclusively on personal protective measures against vector bites. Epidemiologically, OROV maintains two distinct transmission cycles: an urban cycle and a sylvatic cycle [5].

The two distinct transmission cycles of OROV involve specific host-vector systems [6], imposing divergent selective pressures that shape viral genetic diversity. Notably, host-driven selection influences nucleotide usage patterns across OROV genomes. Advances in high-throughput sequencing now enable full-genome characterization, revealing critical insights into viral phylogeny, epidemiology, and genotype classification [7–10]. Like other members of the genus *Orthobunyavirus*, the genome of OROV consists of three segments of single-stranded, negative-sense RNA: a small (S), medium (M), and large (L) segment [7,11]. The S RNA segment encodes the nucleoprotein (also known as the nucleocapsid protein) and a nonstructural protein (NS protein). The M RNA segment encodes a glycoprotein that is cleaved by host proteases into three subunits: Gn, NSm, and Gc. The L RNA segment encodes the viral RdRp. Based on genotypic classification of the S segment in various OROV isolates, four genotypes have been identified [9]. However, nucleotide usage patterns in the S segment inadequately capture the full evolutionary dynamics of the OROV genome due to segment-specific selection constraints.

Nucleotide and dinucleotide composition within coding sequences are nonrandom. Furthermore, the degeneracy of the genetic code allows most amino acids to be encoded by multiple synonymous codons, which exhibit nonrandom, unequal utilization across genomes, a pattern termed synonymous codon usage bias (SCUB). Analyses of nucleotide usage variation in viral coding sequences, particularly for rapidly mutating RNA viruses with short generation times [12], provide valuable insights into viral evolutionary dynamics. Beyond nucleotide sequence alignment, analyses of specific nucleotide arrangements, including dinucleotides, synonymous codons, and flanking nucleotides, elucidate viral evolutionary dynamics in greater detail. Despite increasing reports on OROV evolutionary trends [7,13–15], the underlying mechanisms and patterns of its nucleotide usage variation remain poorly characterized. In this study, we define the evolutionary paradigm of OROV as the integrated profile of selective pressures and mutational biases driving synonymous codon and nucleotide variation in viral proteins. Investigating nucleotide usage in OROV coding sequences elucidates the virus’s evolutionary dynamics during circulation, with implications for molecular diagnostics and vaccine design.

## Materials and methods

### OROV genome information

Based on a collection of OROV genomes available in GenBank (National Center for Biotechnology Information, https://www.ncbi.nlm.nih.gov/), we selected 45 genomes that encompass the virus’s epidemic history (1960–2024) and represent the four genotypes. According to Table S1, we extracted viral coding sequences for the nucleoprotein, glycoprotein, and RdRp from the S, M and L segments, respectively, and quantified the mononucleotide composition in these coding sequences for further estimating the overall nucleotide usage bias.

### Estimation for nucleotide usage bias of viral coding sequences by information entropy

Due to the high mutation rates of RNA viruses, the organization of the viral genome is largely influenced by nucleotide usage variation. In this study, we applied information entropy (Shannon entropy) to assess the extent of nucleotide usage bias resulting from variations in the four mononucleotide compositions. In the information entropy (*H*) formula (1), the four variables correspond to the mononucleotide compositions of adenine (A), guanine (G), uridine (U), and cytosine (C). The term “*N*” refers to the set of four nucleotides, while *p*(*x*) represents the probability of a specific mononucleotide occurring at a given position in the sequence.

(1) $$H=-{\frac{\frac{1}{4}}{\sum }}_{x\in N}p(x)\log p(x)$$

### Estimation for dinucleotide bias of viral coding sequences by dinucleotide odds ratio

To assess dinucleotide bias, the dinucleotide odds ratio was commonly used, expressed as *f*(*xy*)=*f*_*xy*_/*f*_*x*_*f*_*y*_. Where *f*_*xy*_ represents the frequency of the specific nucleotide pair (xy), and *f*_*x*_ and *f*_*y*_ denote the frequencies of nucleotides × and y, respectively. Dinucleotide odds ratio can effectively highlight contrasts between the observed nucleotide pair frequencies and those that are expected from the component mononucleotide frequencies. The dinucleotide odds ratio effectively highlights the differences between the observed frequencies of nucleotide pairs and those expected based on the individual mononucleotide frequencies.

### Analyses for codon usage in viral coding sequences

To elucidate the relationship between nucleotide compositional constraints and natural selective pressures on the overall codon usage pattern in viral coding sequences, a plot was constructed using the effective number of codons (ENC) and the GC content at the third codon position (GC3) [16]. Moreover, the linear regression analysis was used to examine the role of nucleotide compositional constraints, particularly those associated with GC3 content, in shaping the overall codon usage pattern of the target coding sequence.

Furthermore, the relative synonymous codon usage (RSCU) value is a widely used metric for assessing synonymous codon bias in coding sequences [17]. In this study, we used the RSCU formula to quantify the extent of synonymous codon usage bias in the coding sequences of the nucleoprotein, glycoprotein, and RdRp. Given the high mutation rates of RNA virus genomes, the RSCU values for OROV were calculated as the average with a 95% confidence interval. To highlight synonymous codons with significant usage bias, over-represented synonymous codons were defined as those with an RSCU value greater than 1.6, and under-represented codons were defined as those with an RSCU value less than 0.6 [18].

### Estimation for context-dependent codon bias of viral coding sequence

Synonymous codons are not only selected nonrandomly from the synonymous codon family, but the neighboring nucleotides surrounding a codon also influence its selection within the synonymous group. This genetic phenomenon is referred to as context-dependent codon bias (CDCB) [19,20]. Moreover, the nucleotide that most strongly influences CDCB is the first nucleotide following the codon, referred to as the N1 context [21–23]. For the N_1_ context, it refers to the nucleotide immediately following the codon, and is denoted as XYZ_N, where X, Y, Z and N represent any nucleotide. In this study, we examined the extent of CDCB in viral coding sequence by calculating the *R* value. The *R* value represents the relative abundance of a codon (XYZ) with the N_1_ context and is computed as the ratio R_(XYZ_N)_ = F_(XYZ_N)_/F_(XYZ)_F_(N)_, where F_(XYZ_N)_ denotes the frequency of the codon with the N_1_ context, F_(XYZ)_ is the frequency of the codon (XYZ), and F_(XYZ_N)_ is the frequency of nucleotide N in the N_1_ context.

### Mapping evolutionary paradigm of viral coding sequence by principal component analysis

Principal component analysis (PCA) is used to summarize the distance matrix, which represents the pairwise distances between all sample combinations [24]. In this study, we applied the PCA method to map evolutionary paradigm derived from information entropy, dinucleotide odds ratio, and RSCU. When PCA was performed to analyze the overall nucleotide usage bias, the data matrix was constructed using three variables related to the information entropy of the three codon positions in the viral coding sequence. When PCA was performed to estimate nucleotide pair bias, the data matrix was constructed using 16 variables based on the dinucleotide odds ratio in the viral coding sequence. For visualizing synonymous codon usage bias, PCA was applied with a data matrix consisting of 59 variables related to the RSCU in the viral coding sequence. A 3D plot depicting the evolutionary pattern of the OROV target coding sequence was generated using the first three principal components from the PCA.

### Estimating the impact of host selection pressure to OROV evolution

To assess OROV adaptation to human hosts, we calculated three classical adaptation indices: codon adaptation index (CAIcal) [25], relative codon deoptimization index (RCDI) [26] and tRNA adaptation index (tAI) [27–29]. As for assessing the extent of codon usage adaptation of viral coding sequences of OROV to human host, we carried out for the task by means of CAIcal, which is a web-server (http://genomes.urv.es/CAIcal). Moreover, Human (*Homo sapiens*) synonymous codon frequencies were derived from the Codon Usage Database (https://www.kazusa.or.jp/codon/) [30]. The CAIcal values range from 0 to 1, with higher values indicating greater adaptation of exogenous genes to host codon usage patterns [25]. Furthermore, RCDI analysis (http://genomes.urv.cat/CAIcal/RCDI/) was performed to quantify codon deoptimization through comparison of synonymous codon usage between viral genes and the reference host genome [31]. Expression efficiency increases with greater synonymous codon usage similarity between exogenous genes and the host genome [26]. Of note, a low RCDI value could reflect high adaptation of exogenous gene to host. Apart from the genetic role of synonymous codon usage bias in gene expression, protein synthesis is also associated with the waiting time for tRNA correctly entering the ribosomal A site and tRNA concentrations in host cells [32,33]. To assess OROV codon adaptation to humans, we quantified viral synonymous codon usage relative to host tRNA pools using the tAI [28,29]. According to the tAI calculation strategy [27], the related calculating processes were carried out, following the below content.

#### tAI calculation processes

The tAI is a measure of the tRNA usage by coding sequences. To calculate this index, the absolute adaptiveness value *W*_*i*_ for each synonymous codon *i* is defined as the formula (2).

(2) $$W_{i}=\sum_{j=1}^{n_{i}}(1-s_{\mathrm{ij}})\mathrm{tGC}N_{\mathrm{ij}}$$

where *n*_*i*_ is the number of tRNA isoacceptors that recognize the *i* th codon, tGCN_ij_ is the gene copy number of the *j* th tRNA that recognizes the *i* th codon, and *s*_*ij*_ is a selective constraint on the efficiency of the codon-anticodon coupling.

From the *W*_*i*_ values, the relative adaptiveness value *W*_*i*_ of a codon is obtained as formula (3).

(3) $$W_{i}=\left\{\begin{array}{c} W_{i}/W_{\max}\\ W_{\mathrm{mean}} \end{array}\right.\begin{array}{c} \;\mathrm{if}\;W_{i}\neq 0\\ \mathrm{else} \end{array}$$

Where W_max_ is the maximum W_i_ value and W_mean_ is the geometric mean of all W_i_ with W_i_≠0. Finally, the tRNA adaptation index tAI of the target coding sequence is computed as the geometric mean of the relative adaptiveness values of its codons, followed by the formula (4).

(4) $$\mathrm{tAI}=\sqrt[{l}]{\overset{l}{\underset{k=1}{\prod }}W_{\mathrm{ik}}}$$

Where *i*_*k*_ is the codon defined by the *k* th triplet in the target coding sequence and *l* is the length of this sequence in codons (except the stop codons). Consequently, tAI estimates the amount of adaptation of this sequence to its genomic tRNA pool. The tAI values range from 0 to 1. Lower tAI values (approaching 0) indicate reduced translation efficiency, while higher values (approaching 1) denote enhanced efficiency.

### Statistical analysis

The related data in this study were analyzed using a One-way ANOVA, which examines the influence of a single independent variable in the analysis of variance. In One-way ANOVA analyses, the least significance difference (LSD) method, which relies on *t* tests for pariwise comparisons between group averages, was used for post-hoc testing with SPSS 16.0 for Windows. A significant difference was defined as *p* < 0.05.

## Results

### Different extents of nucleotide usage bias in the three codon positions

The coding sequences for nucleoprotein, glycoprotein and RdRp of OROV exhibited a similar organizational pattern in term of nucleotide composition, with U+A content (51%~65%) exceeding G+C content. Despite the comparable nucleotide composition profiles across the three coding sequences, nucleotide usage bias was more pronounced in the glycoprotein and RdRp sequences than in the nucleoprotein sequences (Table 1). Furthermore, the degree of nucleotide usage bias varied across the three codon positions within each coding sequence of OROV (Table 1). Within each viral coding sequence of OROV, nucleotide usage biases was stronger in the first and third codon positions than in the second codon position of the glycoprotein coding sequence, suggesting that nucleotide usage variation in the second codon position is more dynamic than in the other positions; Nucleotide usage biases was stronger in the first and second codon positions than in the third codon position of the nucleoprotein coding sequence, suggesting that nucleotide usage variation in the third position is more dynamic than in the other positions; Nucleotide usage biases was stronger in the second and third codon positions than in the first codon position of the RdRp coding sequence, suggesting that nucleotide usage variation in the first codon position is more dynamic than in the other positions.

**Table 1.** The nucleotide usage bias in OROV coding sequences represented by information entropy.

	N	N1	N2	N3
Glycoprotein	0.482 ± 0.0005	0.477 ± 0.0008	0.482 ± 0.0002	0.477 ± 0.0013
Nucleoprotein	0.499 ± 0.0003	0.487 ± 0.0008	0.489 ± 0.0006	0.498 ± 0.0010
RdRp	0.480 ± 0.0009	0.481 ± 0.0003	0.474 ± 0.0003	0.474 ± 0.0032

The “N” means the overall nucleotide usage bias across the target coding sequence, the “N1,” “N2” and “N3” stand for the nucleotide usage bias in the first, second and third codon sites across the target coding sequence, respectively.

### High levels of genetic diversity of viral coding sequences with respect to nucleotide usage variant

**Figure 1.**
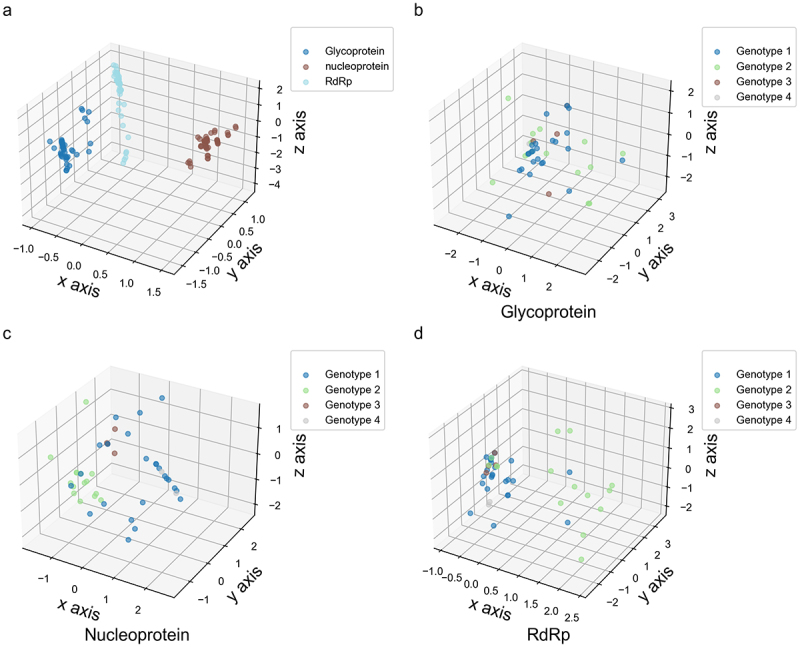
The evolutionary paradigm of nucleotide usage bias in different codon position of OROV coding sequences by PCA method. (a) The 3D-plot for the three viral coding sequences. The × axis accounts for 86.016%, y axis accounts for 13.166% and z axis accounts for 0.818% in PCA. (b) The 3D-plot for OROV glycoprotein coding sequence. The × axis accounts for 38.207%, y axis accounts for 43.601% and z axis accounts for 27.192% in PCA. (c) The 3D-plot for OROV nucleoprotein coding sequence. The × axis accounts for 58.024%, y axis accounts for 31.232% and z axis accounts for 10.744% in PCA. (d) The 3D-plot for OROV RdRp coding sequence. The × axis accounts for 75.540%, y axis accounts for 20.253% and z axis accounts for 4.207% in PCA.

We initially used PCA to analyze the patterns of nucleotide usage variation across different codon positions in the evolutionary development of specific viral proteins. Although nucleotide usage bias at different codon positions may reflect the unique evolutionary features of each viral protein, the coding sequences for each protein did not form distinct clusters (Figure 1(a)), suggesting that OROV owns high mutation rates in its three coding sequences. Subsequently, we applied PCA to assess the genetic diversity of each viral protein with respect to nucleotide usage bias across different codon positions. Generally, nucleotide usage bias at the three codon positions did not exhibit a genotype-specific pattern for each viral protein (Figure 1(b-d)). After comparing the PCA data along the x, y, and z axes, OROV strains isolated from non-human hosts exhibited greater genetic diversity than those isolated from human (Table S2), suggesting that host factor may influence nucleotide usage bias at codon positions in viral proteins. However, no evidence suggests that isolation time is associated with the formation of nucleotide usage bias in the three viral coding sequences (Table S2).

### Strong bias discrepancy of dinucleotide usage in viral coding sequences

**Figure 2.**
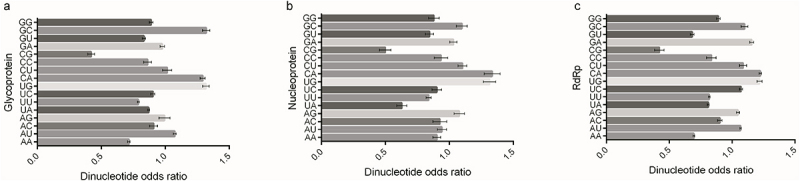
Dinucleotide bias extent of OROV coding sequences. (a) Dinucleotide odds ratios for 16 nucleotide pairs in glycoprotein coding sequence. (b) Dinucleotide odds ratios for 16 nucleotide pairs in nucleoprotein coding sequence. (c) Dinucleotide odds ratios for 16 nucleotide pairs in RdRp coding sequence.

**Figure 3.**
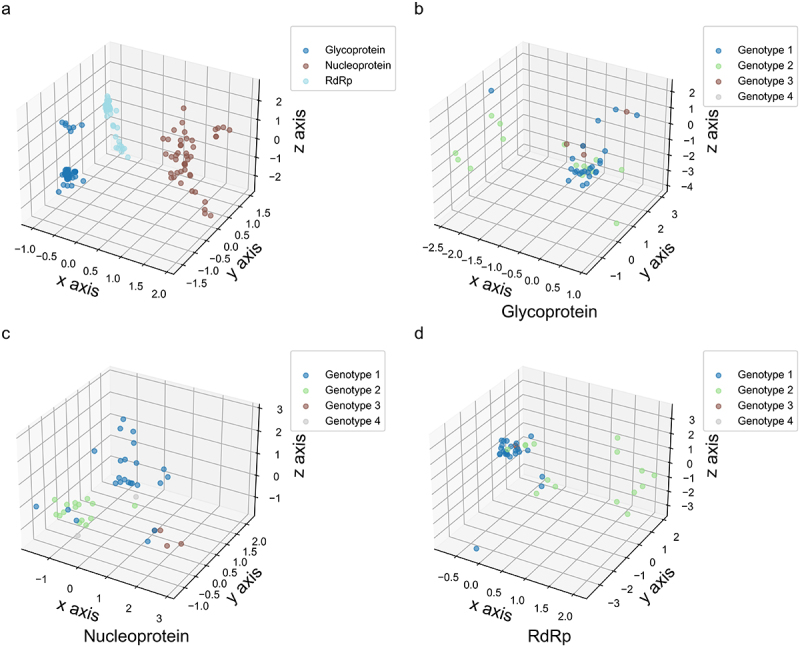
The evolutionary paradigm of dinucleotide bias variants across the whole coding sequence of OROV represented by PCA method. (a) The 3D-plot for the three viral coding sequences. The × axis accounts for 39.755%, y axis accounts for 29.493% and z axis accounts for 10.509% in PCA. (b) The 3D-plot for OROV glycoprotein coding sequence. The × axis accounts for 55.192%, y axis accounts for 18.004% and z axis accounts for 8.750% in PCA. (c) The 3D-plot for OROV nucleoprotein coding sequence. The × axis accounts for 36.131%, y axis accounts for 20.656% and z axis accounts for 15.308% in PCA. (d) The 3D-plot for OROV RdRp coding sequence. The × axis accounts for 58.105%, y axis accounts for 16.023% and z axis accounts for 11.173% in PCA.

**Figure 4.**
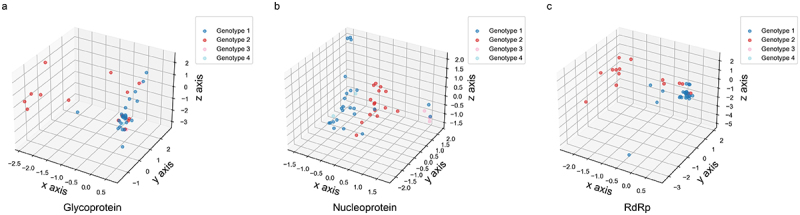
The evolutionary paradigm of dinucleotide bias variants in different codon positions of OROV coding sequences. (a) The 3D-plot for dinucleotide bias variants in different codon positions of glycoprotein coding sequence. The × axis accounts for 54.507%, y axis accounts for 10.895% and z axis accounts for 7.293% in PCA. (b) The 3D-plot for dinucleotide bias variants in different codon positions of nucleoprotein coding sequence. The × axis accounts for 29.749%, y axis accounts for 15.383% and z axis accounts for 13.744% in PCA. (c) The 3D-plot for dinucleotide bias variants in different codon positions of RdRp coding sequence. The × axis accounts for 47.662%, y axis accounts for 15.946% and z axis accounts for 7.959% in PCA.

Due to nucleotide usage bias in the viral coding sequences (Table 1), nucleotide pair usage showed a strong bias (Figure 2). Among the 16 nucleotide pairs, the usage of dinucleotide CpG was most significantly suppressed across the three viral coding sequences (*p* < 0.001). However, unlike the CpG usage pattern in these OROV coding sequences, the dinucleotide UpA was significantly suppressed only in the nucleoprotein coding sequence. Based on the dinucleotide odds ratio data for the viral coding sequences (Figure 2), we further applied PCA to elucidate the evolutionary patterns shaped by dinucleotide bias in these sequences. Although dinucleotde bias distinguished the functional divergence of the three OROV proteins, each protein exhibited a clear discrepancy in nucleotide pair bias (Figure 3(a)), further suggesting that the highly variable nucleotide compositions probably contribute to the evolutionary dynamics driving OROV evolution. Similar to the genetic diversity shown in Figure 1(b-d), dinucleotide bias in the coding sequences of the three proteins did not exhibit a genotype-specific pattern. Additionally, the dinucleotide bias in the coding sequence of the nucleoprotein was significantly weaker than in the glycoprotein and RdRp (Figure 3(b-d)), indicating that the variation of dinucleotide bias in the nucleoprotein coding sequence is more dynamic than in the glycoprotein and RdRp. Furthermore, we applied PCA to illustrate genetic diversity of dinucleotide bias across different codon positions in the three viral coding sequences. Our analysis showed that dinucleotide bias in these positions did not exhibit a genotype-specific pattern (Figure 4), suggesting that the dynamic changes in nucleotide usage influence the usage pattern of the 16 nucleotide pairs in the three coding sequences of OROV.

### Bias discrepancy of synonymous codon usages in viral coding sequences

Based on the strong dinucleotide bias across different codon positions in the viral coding sequences of OROV, we next analyzed the synonymous codon usage patterns in these sequences. The analysis of mean values with 95% confidence interval revealed that the three viral coding sequences contained several synonymous codons with either over-represented or under-represented patterns. In the glycoprotein coding sequence, the over-represented synonymous codons included UUA (L), UCU and UCA (S), CCA (P), ACA (T), GCA (A), and AGA (R). The under-represented codons included CUG and CUC (L), AUC (I), UCC and UCG (S), CCC (P), ACC and ACG (T), GCG (A), UAC (Y), GAG (E), and CGU, CGA, and CGG (R) (Table 2). In the nucleoprotein coding sequence, the over-represented codons included AUU (I), UCA (S), CCA (P), ACU (T), and UGU (C). The under-represented codons owned UUA and CUA (L), CCC (P), ACC and ACG (T), GCG (A), AAA (K), UGC (C), CGA (R), and GGG (G) (Table 3). In the RdRp coding sequence, the over-represented codons had UUA (L), AUA (I), UCU and UCA (S), CCA (P), ACA (T), GCA (A), and AGA and AGG (R). The under-represented codons contained CUC and CUG (L), AUC (I), UCG (S), CCC and CCG (P), ACC and ACG (T), GCC and GCG (A), CAG (Q), AAC (N), and CGU, CGC, CGA and CGG (R), as well as GGC (G) (Table 4). Most synonymous codons containing the dinucleotide CpG were strongly suppressed in usage. Additionally, the three OROV coding sequences exhibited a similar genetic trend, with the number of under-represented synonymous codons exceeding that of over-represented ones. The results indicate that natural selective pressure, which strongly suppresses certain synonymous codon usages, may act as one of the selective forces shaping evolutionary paradigm of OROV.

**Table 2.** Synonymous codon usage variance for OROV glycoprotein.

Synonymous codon (amino acid)	The average RSCU value	95% confidence interval	
		Lower bond	Upper bond
UUU(F)	1.32	1.31	1.33
UUC(F)	0.68	0.67	0.69
**UUA(L)**	1.94	1.87	2.02
UUG(L)	1.15	1.12	1.18
CUU(L)	1.16	1.14	1.17
CUC(L)	0.32	0.29	0.34
CUA(L)	0.88	0.85	0.90
CUG(L)	0.57	0.53	0.60
AUU(I)	1.03	1.01	1.05
AUC(I)	0.43	0.41	0.44
AUA(I)	1.54	1.53	1.56
GUU(V)	1.30	1.26	1.34
GUC(V)	0.82	0.77	0.88
GUA(V)	1.19	1.16	1.23
GUG(V)	0.68	0.64	0.73
**UCU(S)**	1.69	1.67	1.71
UCC(S)	0.34	0.33	0.36
**UCA(S)**	1.68	1.63	1.72
UCG(S)	0.33	0.31	0.36
AGU(S)	1.25	1.20	1.30
AGC(S)	0.70	0.67	0.74
CCU(P)	1.08	1.02	1.15
CCC(P)	0.54	0.51	0.57
**CCA(P)**	1.80	1.77	1.83
CCG(P)	0.58	0.55	0.62
ACU(T)	1.12	1.09	1.15
ACC(T)	0.45	0.44	0.45
**ACA(T)**	2.35	2.30	2.39
ACG(T)	0.08	0.06	0.10
GCU(A)	1.48	1.46	1.49
GCC(A)	0.61	0.59	0.62
**GCA(A)**	1.79	1.77	1.80
GCG(A)	0.14	0.13	0.14
UAU(Y)	1.54	1.50	1.57
UAC(Y)	0.46	0.43	0.50
CAU(H)	1.38	1.37	1.39
CAC(H)	0.62	0.61	0.63
CAA(Q)	1.04	1.00	1.08
CAG(Q)	0.96	0.92	1.00
AAU(N)	1.23	1.20	1.26
AAC(N)	0.77	0.74	0.80
AAA(K)	1.30	1.29	1.32
AAG(K)	0.70	0.68	0.71
GAU(D)	1.28	1.27	1.29
GAC(D)	0.72	0.71	0.73
GAA(E)	1.43	1.41	1.44
GAG(E)	0.57	0.56	0.59
UGU(C)	1.10	1.08	1.11
UGC(C)	0.90	0.89	0.92
CGU(R)	0.25	0.23	0.28
CGC(R)	0.69	0.66	0.72
CGA(R)	0.08	0.04	0.12
CGG(R)	0.35	0.32	0.38
**AGA(R)**	3.20	3.08	3.32
AGG(R)	1.43	1.33	1.53
GGU(G)	1.40	1.37	1.42
GGC(G)	0.94	0.89	0.99
GGA(G)	1.15	1.13	1.18
GGG(G)	0.50	0.44	0.57

The synonymous codons with underline were defined as under-represented codon.

The synonymus codons with bold font were defined as over-represented one.

**Table 3.** Synonymous codon usage variance for OROV nucleoprotein.

Synonymous codon (amino acid)	The average RSCU value	95% confidence interval	
		Lower bond	Upper bond
UUU(F)	0.84	0.82	0.87
UUC(F)	1.16	1.13	1.18
UUA(L)	0.41	0.37	0.45
UUG(L)	1.26	1.19	1.32
CUU(L)	1.02	0.95	1.09
CUC(L)	1.26	1.17	1.34
CUA(L)	0.46	0.38	0.54
CUG(L)	1.61	1.53	1.70
**AUU(I)**	1.97	1.95	1.99
AUC(I)	0.00	^a^—-	^a^—-
AUA(I)	1.03	1.01	1.05
GUU(V)	1.00	0.97	1.04
GUC(V)	1.03	0.98	1.08
GUA(V)	0.86	0.80	0.93
GUG(V)	1.10	1.03	1.18
UCU(S)	1.01	0.92	1.10
UCC(S)	0.92	0.74	1.11
**UCA(S)**	2.11	1.93	2.28
**UCG(S)**	1.85	1.67	2.02
AGU(S)	0.10	0.02	0.18
AGC(S)	^b^0.01	^c^—-	^c^—-
CCU(P)	0.81	0.79	0.83
CCC(P)	0.40	^a^—-	^a^—-
**CCA(P)**	1.99	1.97	2.01
CCG(P)	0.80	^a^—-	^a^—-
**ACU(T)**	2.33	2.29	2.36
ACC(T)	0.55	0.53	0.57
ACA(T)	1.09	1.06	1.12
ACG(T)	0.03	0.00	0.06
GCU(A)	1.63	1.58	1.68
GCC(A)	0.94	0.90	0.98
GCA(A)	1.38	1.32	1.43
GCG(A)	0.05	0.03	0.08
UAU(Y)	0.91	0.85	0.96
UAC(Y)	1.09	1.04	1.15
CAU(H)	1.07	0.95	1.18
CAC(H)	0.93	0.82	1.05
CAA(Q)	1.42	1.37	1.47
CAG(Q)	0.58	0.53	0.63
AAU(N)	0.94	0.88	0.99
AAC(N)	1.06	1.01	1.12
AAA(K)	0.52	0.49	0.56
AAG(K)	1.48	1.44	1.51
GAU(D)	1.10	1.05	1.16
GAC(D)	0.90	0.84	0.95
GAA(E)	0.71	0.68	0.74
GAG(E)	1.29	1.26	1.32
**UGU(C)**	1.89	1.79	1.98
UGC(C)	0.11	0.02	0.21
CGU(R)	1.01	0.95	1.08
CGC(R)	0.83	0.75	0.90
CGA(R)	0.30	0.22	0.37
CGG(R)	0.95	0.89	1.02
AGA(R)	1.67	1.56	1.77
AGG(R)	1.25	1.11	1.39
GGU(G)	1.39	1.33	1.45
GGC(G)	1.06	0.99	1.12
GGA(G)	1.05	1.02	1.09
GGG(G)	0.50	0.46	0.54

^a^means that RSCU value is constant and is excluded from the analysis for 95% confidence interval.

^b^means that all strains with RSCU value = 0, excluding the strain BeH 532,490 with RSCU value = 0.67.

^c^means that RSCU value of AGC(S) was not analyzed by 95% confidence interval.

The synonymous codons with underline were defined as under-represented codon.

The synonymus codons with bold font were defined as over-represented one.

**Table 4.** Synonymous codon usage variance for OROV RdRp.

Synonymous codon (amino acid)	The average RSCU value	95% confidence interval	
		Lower bond	Upper bond
UUU(F)	1.20	1.18	1.22
UUC(F)	0.80	0.78	0.82
**UUA(L)**	2.01	1.98	2.04
UUG(L)	1.13	1.09	1.18
CUU(L)	0.84	0.83	0.86
CUC(L)	0.39	0.38	0.39
CUA(L)	1.11	1.09	1.12
CUG(L)	0.52	0.50	0.53
AUU(I)	0.85	0.85	0.86
AUC(I)	0.42	0.41	0.43
**AUA(I)**	1.73	1.73	1.74
GUU(V)	1.07	1.04	1.09
GUC(V)	0.77	0.73	0.80
GUA(V)	1.44	1.40	1.47
GUG(V)	0.73	0.69	0.76
**UCU(S)**	1.67	1.65	1.70
UCC(S)	0.61	0.58	0.65
**UCA(S)**	1.98	1.94	2.03
UCG(S)	0.23	0.20	0.25
AGU(S)	0.90	0.89	0.91
AGC(S)	0.61	0.60	0.62
CCU(P)	1.52	1.50	1.53
CCC(P)	0.25	0.22	0.27
**CCA(P)**	2.04	2.02	2.07
CCG(P)	0.20	0.18	0.22
ACU(T)	1.19	1.15	1.23
ACC(T)	0.34	0.32	0.36
**ACA(T)**	2.15	2.13	2.18
ACG(T)	0.32	0.31	0.33
GCU(A)	1.16	1.14	1.18
GCC(A)	0.53	0.51	0.54
**GCA(A)**	2.27	2.23	2.30
GCG(A)	0.05	0.03	0.07
UAU(Y)	1.34	1.33	1.36
UAC(Y)	0.66	0.64	0.67
CAU(H)	1.40	1.39	1.41
CAC(H)	0.60	0.59	0.61
CAA(Q)	1.48	1.46	1.50
CAG(Q)	0.52	0.50	0.54
AAU(N)	1.41	1.40	1.42
AAC(N)	0.59	0.58	0.60
AAA(K)	1.36	1.35	1.37
AAG(K)	0.64	0.63	0.65
GAU(D)	1.32	1.31	1.33
GAC(D)	0.68	0.67	0.69
GAA(E)	1.40	1.39	1.41
GAG(E)	0.60	0.59	0.61
UGU(C)	0.84	0.81	0.87
UGC(C)	1.16	1.13	1.19
CGU(R)	0.27	0.26	0.28
CGC(R)	0.24	0.21	0.26
CGA(R)	0.54	0.51	0.57
CGG(R)	0.37	0.35	0.40
**AGA(R)**	2.86	2.81	2.92
**AGG(R)**	1.72	1.66	1.77
GGU(G)	0.91	0.87	0.94
GGC(G)	0.55	0.53	0.58
GGA(G)	1.45	1.43	1.47
GGG(G)	1.08	1.06	1.11

The synonymus codons with bold font were defined as over-represented one.

The synonymous codons with underline were defined as under-represented codon.

**Figure 5.**
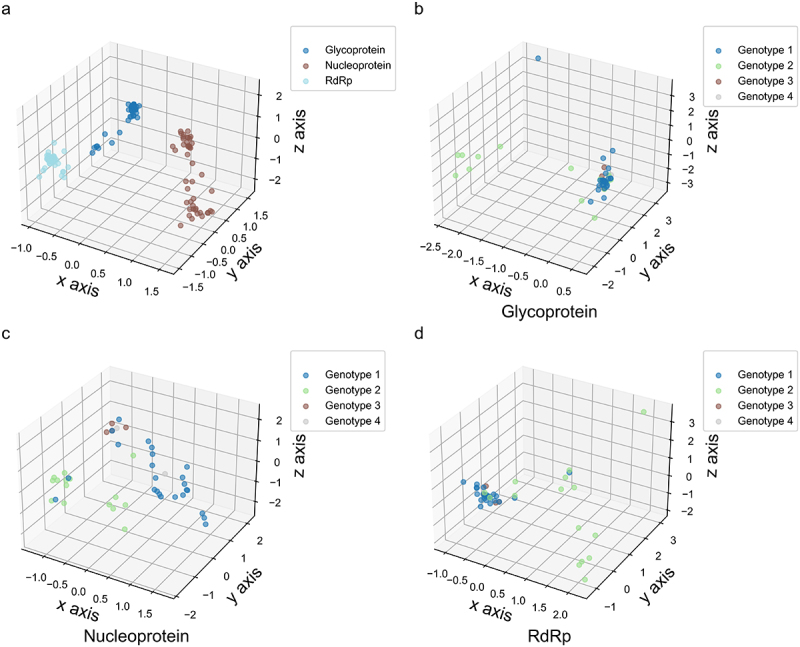
The evolutionary paradigm of RSCU for OROV coding sequences represented by PCA method. (a) The 3D-plot for the three viral coding sequences. The × axis accounts for 57.118%, y axis accounts for 16.859% and z axis accounts for 5.881% in PCA. (b) The 3D-plot for glycoprotein coding sequence. The × axis accounts for 51.933%, y axis accounts for 10.340% and z axis accounts for 7.221% in PCA. (c) The 3D-plot for nucleoprotein coding sequence. The × axis accounts for 22.711%, y axis accounts for 15.046% and z axis accounts for 10.830% in PCA. (d) The 3D-plot for RdRp coding sequence. The × axis accounts for 40.764%, y axis accounts for 12.989% and z axis accounts for 11.330% in PCA.

Based on the evolutionary paradigms of the three coding sequences illustrated by nucleotide usage bias and dinucleotide bias (Figures 1 and 3), PCA was also used to explore the evolutionary paradigm of the viral sequences in relation to synonymous codon usage. As shown in Figure 5, although the synonymous codon usage pattern generally distinguished viral coding sequences with different functions (Figure 5(a)), it failed to exhibit a genotype-specific pattern for the three coding sequences, particularly the coding sequence for nucleoprotein of OROV (Figure 5(b-d)). This further implies that nucleotide composition constraints remain an important evolutionary dynamic in OROV evolution.

### Illustration of the overall codon usage bias for viral coding sequences

**Figure 6.**
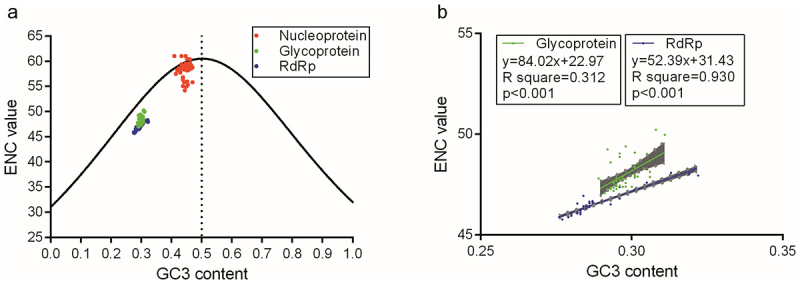
The relationship between ENC and GC3 content in OROV coding sequences. (a) The overall codon usage pattern represented by the plot of enc vs. GC3 content in the three coding sequences of OROV. (b) The linear relationships between enc values and GC3 content in coding sequences for glycoprotein and RdRp of OROV. The gray regions stand for 95% confidential interval.

To directly reflect the roles of nucleotide composition constraints and natural selective pressure in shaping overall codon usage bias, a plot of ENC value versus GC3 content was constructed for the three OROV coding sequences. As shown in Figure 6(a), the overall codon usage patterns of the glycoprotein and RdRp coding sequences differed significantly from that of the nucleoprotein coding sequence of OROV. Compared with the overall codon usage pattern of nucleoprotein, the glycoprotein and RdRp coding sequences fell below the excepted trendline, rather than aligning with it, indicating that natural selective pressure strongly influences the codon usage patterns of these two proteins (Figure 6(a)). Moreover, strong positive correlations were observed between GC3 content and ENC value for glycoprotein and RdRp, whereas no such correlation was observed in the nucleoprotein coding sequence (*p* > 0.05). Additionally, the data points for RdRp better fit a linear regression model (R^2^ = 0.930) compared to those for glycoprotein (R^2^ = 0.312) (Figure 6(b)), suggesting that the nucleotide composition constraint associated with GC3 content plays a more significant role in RdRp than in glycoprotein.

### Strong bias discrepancy of context-dependent codon bias for viral coding sequences

Based on data related to nucleotide usage bias, dinucleotide bias, and synonymous codon usage bias in the viral coding sequences of OROV, we further quantified the *R* values for the three coding sequences to estimate the extent of CDCB for each synonymous codon. The *R* values for each viral protein varied in a relatively broad range (Table S3), indicating a bias discrepancy in synonymous codons adjacent to the next nucleotide type in the OROV coding sequences. Among the synonymous codons with favorable and unfavorable nucleotide (Table 5), the model XYC_G (where X and Y represent nucleotides) appeared only in the group of codons with unfavorable nucleotides, while the model XYU_A was predominantly found in this group, except for UGU_A, which was listed in the group of codons with favorable nucleotides. The patterns of CDCB in the three viral coding sequences further suggest that natural selective pressure plays a significant role in the evolutionary paradigm of OROV, particularly in relation to nucleotide pair and synonymous codon usage.

**Table 5.** Synonymous codons with significant context-dependent codon bias in coding sequences for glycoprotein, nucleoprotein and RdRp of OROV.

Coding sequence	Codon neighboring the favorable nucleotide	Codon neighboring the unfavorable nucleotide
Glycoprotein	AAA_G (1.508 ± 0.041)	AAA_A(0.706 ± 0.041)
	AAC_A (1.364 ± 0.041)	AAC_G (0.368 ± 0.100)
	AAG_A (1.251 ± 0.032)	AAG_G (0.486 ± 0.071)
	ACU_G (1.597 ± 0.208)	ACU_C (0.600 ± 0.092)
	ACG_G (1.392 ± 0.374)	ACG_U (0.577 ± 0.197)
	AGU_G (1.567 ± 0.210)	AGU_A (0.686 ± 0.075)
	AGC_A (1.358 ± 0.068)	AGC_G (0.082 ± 0.166)
	UAC_A (1.604 ± 0.068)	UAC_G (0.298 ± 0.113)
	UAG_A (1.461 ± 0.115)	UAG_C (0.471 ± 0.197)
	UUC_A (1.271 ± 0.075)	UUC_G (0.300 ± 0.072)
	UCU_G (1.699 ± 0.089)	UCU_C (0.482 ± 0.089)
	UGC_C (1.243 ± 0.105)	UGC_G (0.413 ± 0.094)
	CAC_C (1.437 ± 0.182)	CAC_G (0.058 ± 0.085)
	CUA_U (1.340 ± 0.055)	CUA_C (0.570 ± 0.124)
	CUU_G (1.760 ± 0.187)	CUU_A (0.702 ± 0.066)
	CUC_U (1.353 ± 0.215)	CUC_G (0.034 ± 0.102)
	CCG_C (2.269 ± 0.696)	CCG_G (0.394 ± 0.255)
	CGA_A (1.349 ± 0.144)	CGA_G (0.323 ± 0.133)
	CGC_A (1.427 ± 0.219)	CGC_G (0.000 ± 0.000)
	GAC_A (1.421 ± 0.124)	GAC_G (0.357 ± 0.105)
	GCU_G (1.684 ± 0.070)	GCU_A (0.584 ± 0.058)
	GGU_U (1.334 ± 0.092)	GGU_A (0.619 ± 0.076)
Nucleoprotein	AAG_A (1.852 ± 0.217)	AAG_U (0.479 ± 0.074)
	AUU_U (1.766 ± 0.271)	AUU_A (0.349 ± 0.030)
	ACA_U (1.733 ± 0.198)	ACA_C (0.015 ± 0.070)
	ACU_U (1.863 ± 0.127)	ACU_A (0.417 ± 0.070)
	ACC_A (1.673 ± 0.178)	ACC_G (0.399 ± 0.064)
	AGG_C (1.771 ± 0.288)	AGG_U (0.544 ± 0.162)
	UUA_C (1.970 ± 0.252)	UUA_U (0.388 ± 0.160)
	UUC_C (1.441 ± 0.210)	UUC_G (0.377 ± 0.122)
	UUG_A (1.394 ± 0.101)	UUG_U (0.285 ± 0.124)
	UCU_U (1.802 ± 0.177)	UCU_A (0.391 ± 0.157)
	UGU_A (1.641 ± 0.164)	UGU_G (0.491 ± 0.198)
	CAU_U (1.630 ± 0.215)	CAU_G (0.666 ± 0.153)
	CAC_U (1.512 ± 0.254)	CAC_G (0.000 ± 0.000)
	CCA_A (1.469 ± 0.163)	CCA_U (0.472 ± 0.028)
	CCU_U (1.831 ± 0.425)	CCU_A (0.417 ± 0.170)
	CCC_A (2.478 ± 0.324)	CCC_G (0.043 ± 0.140)
	GAU_G (2.315 ± 0.269)	GAU_C (0.463 ± 0.161)
	GUG_C (1.660 ± 0.226)	GUG_U (0.392 ± 0.165)
	GCU_G (2.661 ± 0.183)	GCU_U (0.297 ± 0.158)
	GCC_A (1.802 ± 0.125)	GCC_C (0.423 ± 0.138)
	GGA_C (1.596 ± 0.382)	GGA_G (0.324 ± 0.097)
	GGC_C (1.999 ± 0.281)	GGC_G (0.019 ± 0.073)
RdRp	AAA_U (1.360 ± 0.020)	AAA_A (0.593 ± 0.036)
	AUU_U (1.333 ± 0.050)	AUU_A (0.655 ± 0.052)
	ACC_C (1.472 ± 0.355)	ACC_G (0.335 ± 0.134)
	ACG_A (1.438 ± 0.058)	ACG_A (0.534 ± 0.082)
	AGC_A (1.418 ± 0.064)	AGC_G (0.321 ± 0.090)
	UAC_A (1.269 ± 0.046)	UAC_G (0.320 ± 0.047)
	UAG_A (1.320 ± 0.048)	UAG_U (0.603 ± 0.075)
	UUU_C (1.480 ± 0.042)	UUU_A (0.661 ± 0.031)
	UCC_C (1.307 ± 0.395)	UCC_G (0.213 ± 0.107)
	UGU_C (1.818 ± 0.130)	UGU_U (0.755 ± 0.064)
	UGC_C (1.494 ± 0.089)	UGC_G (0.310 ± 0.055)
	CCA_G (1.496 ± 0.068)	CCA_C (0.567 ± 0.055)
	CCU_G (1.646 ± 0.094)	CCU_A (0.691 ± 0.039)
	GAC_A (1.342 ± 0.063)	GAC_G (0.393 ± 0.096)
	GUC_U (1.426 ± 0.111)	GUC_G (0.329 ± 0.129)
	GCC_A (1.279 ± 0.082)	GCC_G (0.489 ± 0.104)
	GCG_G (2.167 ± 0.616)	GCG_U (0.438 ± 0.176)
	GGC_A (1.284 ± 0.069)	GGC_G (0.444 ± 0.123)

The data are composed of the mean value ± the standard errors.

### Graded adaptation of viral codon usage to the human host

**Figure 7.**
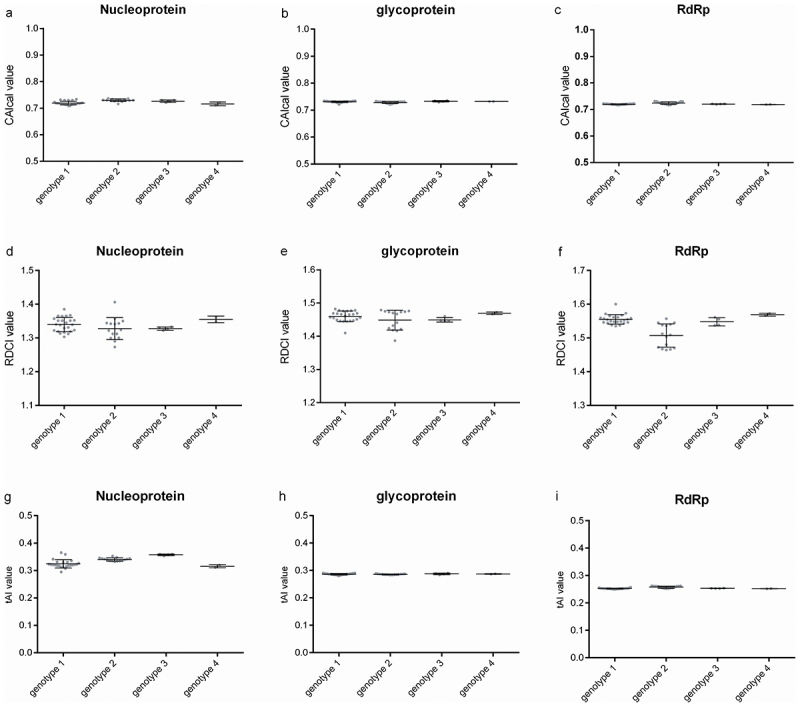
The relationships between OROV and human host with respect to codon usage and tRNA adaptation. (a) The CAIcal value for coding sequence of nucleoprotein within four genotypes. (b) The CAIcal value for coding sequence of glycoprotein within four genotypes. (c) The CAIcal value for coding sequence of RdRp within four genotypes. (d) The RDCI value for coding sequence of nucleoprotein within four genotypes. (e) The RDCI value for coding sequence of glycoprotein within four genotypes. (f) The RDCI value for coding sequence of RdRp within four genotypes. (g) The tAI value for coding sequence of nucleoprotein within four genotypes. (h) The tAI value for coding sequence of glycoprotein within four genotypes. (i) The tAI value for coding sequence of RdRp within four genotypes.

The CAIcal measure, which is popular to estimate codon adaptation of the target gene to host, reflected that the similar codon adaptations of the three viral coding sequences of OROV to human host (Figure 7(a-c)). As for the four genotypes of OROV, the CAIcal values of the coding sequences of nucleoprotein ranged from 0.709–0.737 (Figure 7(a)); the values of the coding sequences of glycoprotein ranged from 0.721–0.735 (Figure 7(b)); the values of the coding sequences of RdRp ranged from 0.716–0.731 (Figure 7(c)). The results suggest significant codon adaptation of all three OROV coding sequences to the human host. Notably, RCDI values of the three coding sequence of OROV exhibited different extents, namely RCDI values corresponding to coding sequences of nucleoprotein ranging from 1.273 to 1.406, those of glycoprotein ranging from 1.387 to 1.406 and those of RdRp ranging from 1.464 to 1.600. According to the RCDI evaluation criterion [26], the three OROV coding sequences across all genotypes exhibited suboptimal translation efficiency in human hosts, as their RCDI values significantly deviated from 1 (Figure 7(d-f)). Furthermore, the tAI measure was employed to assess the codon adaptation of the three coding sequences of OROV to human host with translation rate. As a well-established metric for predicting the translation efficiency of exogenous genes, the tAI values for the three coding sequences of OROV obviously displayed different extents of translation rate in human host (Figure 7(g-i)). Comparative analysis of tAI values among the three OROV coding sequences across four genotypes revealed significant differences in translational efficiency. The nucleoprotein coding sequence with the tAI values ranging from 0.295 to 0.365 displayed the highest translation rates, followed by coding sequences for glycoprotein with tAI values ranging from 0.281 to 0.291, the coding sequence for RdRp with tAI values ranging from 0.249 to 0.262 displayed the lowest translation rate (Figure 7(g-i)). Collectively, the three coding sequences of OROV (an arbovirus) owned the relatively low levels of translation rate in human host.

## Discussion

Sequence data related to nucleotide usage variation are widely used to investigate genetic relationships among organisms. In the evolutionary paradigm of viruses, nucleotide usage pattern are nonrandom, and synonymous codon usage and codon order also exhibit nonrandom patterns [9,34]. A previous report indicated divergent evolutionary rates across the three OROV segments: segment S with 1.75 × 10^−3^ substitutions/site/year, segment M with 3.65 × 10^−4^ substitutions/site/year, and segment L with 1.64 × 10^−3^ substitutions/site/year [6]. In the three OROV coding sequences, the models of nucleotide usage bias, dinucleotide bias, and codon usage bias did not reflect genotype-specific characteristics but instead revealed distinct evolutionary paradigms across the viral coding sequences, covering the majority of regions in the three OROV genome segments (Figures 1,3–5). The genetic feature is likely explained by the involvement of protein-function-dependent evolutionary dynamics in the evolutionary paradigms of the corresponding proteins. Nucleotide and codon usage biases are often influenced by factors related to species or protein functions [34]. These biases are driven by two major factors: natural selective pressure from the host and nucleotide composition constraints imposed by mutation pressure in viral life cycle. Natural selective pressure has multiple drivers, including host proteins that recognize specific nucleotide patterns, which, when triggered, induce mutations or activate intrinsic or innate immune responses. For example, the nucleoprotein encoded by the S segment and the RdRp work together to replicate the viral genome, with each segment potentially restricting the molecular evolution of the other. In contrast, the M segment, which encodes viral glycoproteins, may be more prone to mutations due to higher natural selective pressure in its coding region, as these proteins are major determinants of host range [35]. The associated viral proteins enable OROV to replicate efficiently in host cells and to evade or resist host immune responses. Additionally, both efficient viral genome replication and immune resistant are strongly linked to the nucleotide pair compositions of the viral genome [36–39]. Despite the broad variation in dinucleotide bias across the three OROV coding sequences (Figures 3 and 4), the dinucleotide CpG was predominantly suppressed in usage for the corresponding viral proteins (Figure 2), strongly suggesting that translation selection driven by host cell factors associated with gene replication and immune response plays a key role in the evolutionary paradigm of OROV. A previous study indicated that OROV infection and replication can persist in human Jeko-1 Mantle lymphoma B cells, Jurkat T cells, and THP-1 monocytic cells [40]. Moreover, the dinucleotide relative abundance deviations shown in Figure 2 likely reflect base-step stacking capacities and higher-order RNA folding features. This may be due to base-step conformational preferences, which are reflected in parameters such as slide, roll, tilt, propeller twist, and helical twist [41–44]. Additionally, dinucleotide composition-dependent RNA/DNA structural features strongly influence protein-RNA interactions [45–47].

Frequent genomic reassortment events in OROV likely represent a key evolutionary driver, generating diverse evolutionary trajectories through segment recombination and host adaptation [48–51]. As shown in Tables 2–4, the over-represented and under-represented synonymous codons reflect translation selection involved in shaping the overall codon usage pattern of OROV, strongly indicating that natural selective pressure plays a key role in the synonymous codon usage of OROV. The distinct codon usage patterns observed in OROV, including significant suppression of CpG-containing synonymous codons and context-dependent codon preferences, may reflect viral strategies to evade host immune surveillance. Specifically, these patterns may facilitate viral escape from recognition by the Zinc-finger antiviral protein (ZAP), which selectively targets CpG-rich RNA sequences [38,39]. Moreover, biased codon usage directly regulates translational efficiency and viral protein expression levels [35,52], which has implications for synthetic biology applications. These findings provide a rational basis for engineering attenuated viral vaccines or optimizing viral vectors through targeted codon recoding, as evidenced by successful applications of codon-pair deoptimization strategies in other RNA viruses [53,54]. Generally, translational selection or codon usage bias is more observed in unicellular organisms (including viruses) than in eukaryotes, where a specific subset of synonymous codons is closely linked to translation efficiency or the fine-tuning translation [52,55,56]. Apart from synonymous codon usage bias in the three viral proteins of OROV, neighboring nucleotides flanking a codon influenced the selection of that particular codon from the synonymous family (Table 5), strongly suggesting that the bias of neighboring synonymous codon can affect viral protein function and pathogenesis. This genetic phenomenon has been observed in the genomes of various organisms (including viruses and microorganisms) and is explained as a direct consequence of dinucleotide bias [22,53,54,57–60]. Beyond the influence of dinucleotide usage bias on synonymous codon usage bias, accumulating evidence indicates that codon pair bias does not independently reflect host translational preferences. Rather, this phenomenon emerges as an epiphenomenon of underlying dinucleotide usage bias, particularly the pronounced suppression of CpG and TpA dinucleotides, which is prevalent across many vertebrate viruses [57]. Notably, dinucleotide usage bias, particularly CpG and TpA, plays a more important role in regulating virus fitness than codon pair bias [61].

Although significant deviations in overall codon usage were observed for the three coding sequences of OROV, the usage pattern for nucleoprotein differed markedly from those for glycoprotein and RdRp (Figure 6), suggesting that the fine-tune translational selection may regulate gene expressions in these coding sequences of OROV in different ways. Based on the relationships between ENC and GC3 content in different viruses, the evolutionary paradigm represented by the overall codon usage pattern has been considered a new pathway for reflecting gene expression through alternative regulatory mechanisms [9,62,63]. The evolutionary paradigm of OROV is likely driven by a complex interplay of selective forces associated with the viral life cycle and the signature of the OROV genome [64–66]. Comparative analysis of the CAIcal for OROV coding sequences in human hosts (Figure 7(a-c)), suggests that the RCDI may serve as a more robust metric for evaluating host-virus coevolutionary adaptation (Figure 7(d-f)). A high RCDI (more than 1) might imply suboptimal adaptation of OROV coding sequences to the human host in the terms of synonymous codon usage. This observed codon deoptimization may consequently lead to reduced translational efficiency of viral genes in the host system. The precise molecular mechanisms underlying tRNA abundance-mediated codon adaptation in host-virus systems remain largely elusive, representing a fundamental unresolved question in viral evolution research [29,67,68]. The RCDI-based analysis of OROV’s three coding sequences consistently indicates suboptimal translational efficiency in human hosts, congruent with their codon deoptimization patterns (Figure 7(g-i)).

## Conclusion

Given the substantial genetic diversity observed among OROV proteins, we systematically characterized nucleotide composition, dinucleotide frequencies, synonymous codon usage, and flanking nucleotide contexts across three principal coding regions of the OROV genome. Our analyses reveal that nucleotide and dinucleotide usage biases exhibit protein function-dependent distributions, independent of genotype classification. Consistent with these observations, synonymous codon usage and context-dependent codon preferences predominantly reflect functional constraints imposed by viral protein requirements and host-pathogen interactions, rather than phylogenetic relationships. These genomic biases, particularly CpG dinucleotide suppression and nonrandom codon context arrangements, are evolutionarily constrained by both host immune surveillance and translational selection pressures. Notably, the nucleoprotein exhibits significantly stronger evolutionary constraints than glycoprotein and RdRp, with the latter two proteins demonstrating greater susceptibility to mutational bias and compositional drift. As an arbovirus, OROV exhibits suboptimal translational efficiency in human hosts, as evidenced by consistently low codon adaptation indices across its coding sequences its coding sequences. Beyond their evolutionary implications, these findings possess substantial translational relevance: the conserved codon and dinucleotide signatures identified in glycoprotein and RdRp regions may facilitate development of sequence-based diagnostic assays and enhance viral surveillance capabilities. Furthermore, the elucidated codon usage profiles establish a conceptual framework for implementing rational genome recoding approaches in synthetic vaccinology and viral vector engineering.

## Supplementary Material

Table S3 Context dependent codon bias for three segments of OROV_.xls

Table S2 The x y and z axes data of PCA for nucleotide usage bias in different codon positions of viral coding sequences_.xls

Table S1 Information about OROV.doc

## Data Availability

The data supporting the findings of this study are available within the article and the data that support the findings of this study are available in Science Data Bank at: https://doi.org/10.57760/sciencedb.17237.

## References

[1] Morrison A, White JL, Hughes HR, et al. Oropouche virus disease among U.S. travelers - United States, 2024. MMWR Morb Mortal Wkly Rep. 2024;73(35):769–16. doi: 10.15585/mmwr.mm7335e1 39236058 PMC11376504

[2] Benitez AJ, Alvarez M, Perez L, et al. Oropouche fever, Cuba, May 2024. Emerg Infect Dis. 2024;30(10):2155–2159. doi: 10.3201/eid3010.240900 39255237 PMC11431908

[3] Guagliardo SAJ, Connelly CR, Lyons S, et al. Reemergence of Oropouche virus in the Americas and risk for spread in the United States and its territories, 2024. Emerg Infect Dis. 2024;30(11):2241–2249. doi: 10.3201/eid3011.241220 39353409 PMC11521165

[4] Branda F, Ciccozzi M, Scarpa F. Oropouche virus presenting in Italy after travel to Cuba. New Microbes New Infect. 2024;60–61:101450. doi: 10.1016/j.nmni.2024.101450 PMC11295918 39100738

[5] Romero-Alvarez D, Escobar LE. Oropouche fever, an emergent disease from the Americas. Microbes Infect. 2018;20(3):135–146. doi: 10.1016/j.micinf.2017.11.013 29247710

[6] Gutierrez B, Wise EL, Pullan ST, et al. Evolutionary dynamics of Oropouche virus in South America. J Virol. 2020;94(5):e01127–19. doi: 10.1128/JVI.01127-19 31801869 PMC7022353

[7] da Rosa JFT, de Souza WM, de Paula Pinheiro F, et al. Oropouche virus: clinical, epidemiological, and molecular aspects of a neglected orthobunyavirus. Am J Trop Med Hyg. 2017;96(5):1019–1030. doi: 10.4269/ajtmh.16-0672 28167595 PMC5417190

[8] Ciuoderis KA, Berg MG, Perez LJ, et al. Oropouche virus as an emerging cause of acute febrile illness in Colombia. Emerg Microbes Infect. 2022;11(1):2645–2657. doi: 10.1080/22221751.2022.2136536 36239235 PMC9639516

[9] Vasconcelos HB, Nunes MRT, Casseb LMN, et al. Molecular epidemiology of Oropouche virus, Brazil. Emerg Infect Dis. 2011;17(5):800–806. doi: 10.3201/eid1705.101333 21529387 PMC3321770

[10] Nunes MRT, de Souza WM, Savji N, et al. Oropouche orthobunyavirus: genetic characterization of full-length genomes and development of molecular methods to discriminate natural reassortments. Infect Genet Evol. 2019;68:16–22. doi: 10.1016/j.meegid.2018.11.020 30504003

[11] Vasconcelos HB, Azevedo RSS, Casseb SM, et al. Oropouche fever epidemic in northern Brazil: epidemiology and molecular characterization of isolates. J Clin Virol. 2009;44(2):129–133. doi: 10.1016/j.jcv.2008.11.006 19117799

[12] Duffy S, Shackelton LA, Holmes EC. Rates of evolutionary change in viruses: patterns and determinants. Nat Rev Genet. 2008;9(4):267–276. doi: 10.1038/nrg2323 18319742

[13] Wesselmann KM, Postigo-Hidalgo I, Pezzi L, et al. Emergence of Oropouche fever in Latin America: a narrative review. Lancet Infect Dis. 2024;24(7):e439–e452. doi: 10.1016/S1473-3099(23)00740-5 38281494

[14] Moreira HM, Sgorlon G, Queiroz JAS, et al. Outbreak of Oropouche virus in frontier regions in western Amazon. Microbiol Spectr. 2024;12(3):e0162923. doi: 10.1128/spectrum.01629-23 38323826 PMC10913433

[15] Tilston-Lunel NL. Oropouche virus: an emerging orthobunyavirus. J Gen Virol. 2024;105(10):002027. doi: 10.1099/jgv.0.002027 39351896 PMC11443551

[16] Wright F. The „effective number of codons“ used in a gene. Gene. 1990;87(1):23–29. doi: 10.1016/0378-1119(90)90491-9 2110097

[17] Sharp PM, Li WH. Codon usage in regulatory genes in Escherichia coli does not reflect selection for „rare“ codons. Nucleic Acids Res. 1986;14(19):7737–7749. doi: 10.1093/nar/14.19.7737 3534792 PMC311793

[18] Zhou J-H, Gao Z-L, Zhang J, et al. The analysis of codon bias of foot-and-mouth disease virus and the adaptation of this virus to the hosts. Infect Genet Evol. 2013;14:105–110. doi: 10.1016/j.meegid.2012.09.020 23220329

[19] Yarus M, Folley LS. Sense codons are found in specific contexts. J Mol Biol. 1985;182(4):529–540. doi: 10.1016/0022-2836(85)90239-6 3892014

[20] Gouy M. Codon contexts in enterobacterial and coliphage genes. Mol Biol Evol. 1987;4(4):426–444. doi: 10.1093/oxfordjournals.molbev.a040450 3128715

[21] Fedorov A, Saxonov S, Gilbert W. Regularities of context-dependent codon bias in eukaryotic genes. Nucleic Acids Res. 2002;30(5):1192–1197. doi: 10.1093/nar/30.5.1192 11861911 PMC101244

[22] Berg OG, Silva PJ. Codon bias in Escherichia coli: the influence of codon context on mutation and selection. Nucleic Acids Res. 1997;25(7):1397–1404. doi: 10.1093/nar/25.7.1397 9060435 PMC146607

[23] Karlin S, Mrázek J. What drives codon choices in human genes? J Mol Biol. 1996;262(4):459–472. doi: 10.1006/jmbi.1996.0528 8893856

[24] Konishi T, Matsukuma S, Fuji H, et al. Principal component analysis applied directly to sequence matrix. Sci Rep. 2019;9(1):19297. doi: 10.1038/s41598-019-55253-0 31848355 PMC6917774

[25] Puigbò P, Bravo IG, Garcia-Vallve S. Caical: a combined set of tools to assess codon usage adaptation. Biol Direct. 2008;3(1):38. doi: 10.1186/1745-6150-3-38 18796141 PMC2553769

[26] Mueller S, Papamichail D, Coleman JR, et al. Reduction of the rate of poliovirus protein synthesis through large-scale codon deoptimization causes attenuation of viral virulence by lowering specific infectivity. J Virol. 2006;80(19):9687–9696. doi: 10.1128/JVI.00738-06 16973573 PMC1617239

[27] dos Reis M, Savva R, Wernisch L. Solving the riddle of codon usage preferences: a test for translational selection. Nucleic Acids Res. 2004;32(17):5036–5044. doi: 10.1093/nar/gkh834 15448185 PMC521650

[28] dos Reis M, Wernisch L, Savva R. Unexpected correlations between gene expression and codon usage bias from microarray data for the whole Escherichia coli K-12 genome. Nucleic Acids Res. 2003;31(23):6976–6985. doi: 10.1093/nar/gkg897 14627830 PMC290265

[29] Duret L. tRNA gene number and codon usage in the C. elegans genome are co-adapted for optimal translation of highly expressed genes. Trends Genet. 2000;16(7):287–289. doi: 10.1016/s0168-9525(00)02041-2 10858656

[30] Nakamura Y, Gojobori T, Ikemura T. Codon usage tabulated from international DNA sequence databases: status for the year 2000. Nucleic Acids Res. 2000;28(1):292. doi: 10.1093/nar/28.1.292 10592250 PMC102460

[31] Puigbò P, Aragonès L, Garcia-Vallvé S. Rcdi/ercdi: a web-server to estimate codon usage deoptimization. BMC Res Notes. 2010;3(1):87. doi: 10.1186/1756-0500-3-87 20356391 PMC2853550

[32] Varenne S, Buc J, Lloubes R, et al. Translation is a non-uniform process. Effect of tRNA availability on the rate of elongation of nascent polypeptide chains. J Mol Biol. 1984;180(3):549–576. doi: 10.1016/0022-2836(84)90027-5 6084718

[33] Sørensen MA, Kurland CG, Pedersen S. Codon usage determines translation rate in Escherichia coli. J Mol Biol. 1989;207(2):365–377. doi: 10.1016/0022-2836(89)90260-x 2474074

[34] Gaunt ER, Digard P. Compositional biases in RNA viruses: causes, consequences and applications. Wiley Interdiscip Rev RNA. 2022;13(2):e1679. doi: 10.1002/wrna.1679 34155814 PMC8420353

[35] Zhang Y, Liu X, Wu Z, et al. Oropouche virus: a neglected global arboviral threat. Virus Res. 2024;341:199318. doi: 10.1016/j.virusres.2024.199318 38224842 PMC10827532

[36] Roy CN, Benitez Moreno MA, Kline C, et al. Cg dinucleotide removal in bioluminescent and fluorescent reporters improves HIV-1 replication and reporter gene expression for dual imaging in humanized mice. J Virol. 2021;95(19):e0044921. doi: 10.1128/JVI.00449-21 34232063 PMC8428378

[37] Jordan-Paiz A, Franco S, Martinez MA. Reducing HIV-1 env gene CpG frequency increases the replication capacity of the HXB2 virus strain. Virus Res. 2022;310:198685. doi: 10.1016/j.virusres.2022.198685 35041864

[38] Lin Y-T, Chau L-F, Coutts H, et al. Does the zinc finger antiviral protein (ZAP) shape the evolution of herpesvirus genomes? Viruses. 2021;13(9):1857. doi: 10.3390/v13091857 34578438 PMC8473364

[39] Jordan-Paiz A, Franco S, Martinez MA. Synonymous codon pair recoding of the HIV-1 env gene affects virus replication capacity. Cells. 2021;10(7):1636. doi: 10.3390/cells10071636 34209946 PMC8304268

[40] Ribeiro Amorim M, Cornejo Pontelli M, Fabiano de Souza G, et al. Oropouche virus infects, persists and induces IFN response in human peripheral blood mononuclear cells as identified by RNA PrimeFlow^TM^ and qRT-PCR assays. Viruses. 2020;12(7):785. doi: 10.3390/v12070785 32708342 PMC7411765

[41] Kole K, Gupta AM, Chakrabarti J. Conformational stability and order of Hoogsteen base pair induced by protein binding. Biophys Chem. 2023;301:107079. doi: 10.1016/j.bpc.2023.107079 37523944

[42] Zoli M. Base pair fluctuations in helical models for nucleic acids. J Chem Phys. 2021;154(19):194102. doi: 10.1063/5.0046891 34240895

[43] Jensen EA, Allen BD, Kishi Y, et al. Conformational analysis of a covalently cross-linked Watson-Crick base pair model. Bioorg Med Chem Lett. 2008;18(22):5884–5887. doi: 10.1016/j.bmcl.2008.07.113 18706810 PMC2590868

[44] Zacharias M. Base-pairing and base-stacking contributions to double-stranded DNA formation. J Phys Chem B. 2020;124(46):10345–10352. doi: 10.1021/acs.jpcb.0c07670 33156627

[45] Wei J, Chen S, Zong L, et al. Protein-RNA interaction prediction with deep learning: structure matters. Brief Bioinform. 2022;23(1):bbab540. doi: 10.1093/bib/bbab540 34929730 PMC8790951

[46] Corley M, Burns MC, Yeo GW. How RNA-binding proteins interact with RNA: molecules and mechanisms. Mol Cell. 2020;78(1):9–29. doi: 10.1016/j.molcel.2020.03.011 32243832 PMC7202378

[47] Sanchez de Groot N, Armaos A, Graña-Montes R, et al. Rna structure drives interaction with proteins. Nat Commun. 2019;10(1):3246. doi: 10.1038/s41467-019-10923-5 31324771 PMC6642211

[48] Aguilar PV, Barrett AD, Saeed MF, et al. Iquitos virus: a novel reassortant orthobunyavirus associated with human illness in Peru. PLoS Negl Trop Dis. 2011;5(9):e1315. doi: 10.1371/journal.pntd.0001315 21949892 PMC3176741

[49] Navarro J-C, Giambalvo D, Hernandez R, et al. Isolation of Madre de Dios virus (Orthobunyavirus; Bunyaviridae), an Oropouche virus species reassortant, from a monkey in Venezuela. Am J Trop Med Hyg. 2016;95(2):328–338. doi: 10.4269/ajtmh.15-0679 27215299 PMC4973178

[50] Tilston-Lunel NL, Shi X, Elliott RM, et al. The potential for reassortment between Oropouche and Schmallenberg orthobunyaviruses. Viruses. 2017;9(8):220. doi: 10.3390/v9080220 28800086 PMC5580477

[51] Beaty BJ, Bishop DH. Bunyavirus-vector interactions. Virus Res. 1988;10(4):289–301. doi: 10.1016/0168-1702(88)90071-8 3046165

[52] Santos MAS, Moura G, Massey SE, et al. Driving change: the evolution of alternative genetic codes. Trends Genet. 2004;20(2):95–102. doi: 10.1016/j.tig.2003.12.009 14746991

[53] Vaz PK, Armat M, Hartley CA, et al. Codon pair bias deoptimization of essential genes in infectious laryngotracheitis virus reduces protein expression. J Gen Virol. 2023;104(4):104. doi: 10.1099/jgv.0.001836 37010948

[54] Eschke K, Trimpert J, Osterrieder N, et al. Attenuation of a very virulent Marek’s disease herpesvirus (MDV) by codon pair bias deoptimization. PLoS Pathog. 2018;14(1):e1006857. doi: 10.1371/journal.ppat.1006857 29377958 PMC5805365

[55] Sau K, Gupta SK, Sau S, et al. Studies on synonymous codon and amino acid usage biases in the broad-host range bacteriophage kvp40. J Microbiol. 2007;45:58–63. 17342057

[56] Musto H, Cruveiller S, D’Onofrio G, et al. Translational selection on codon usage in Xenopus laevis. Mol Biol Evol. 2001;18(9):1703–1707. doi: 10.1093/oxfordjournals.molbev.a003958 11504850

[57] Kunec D, Osterrieder N. Codon pair bias is a direct consequence of dinucleotide bias. Cell Rep. 2016;14(1):55–67. doi: 10.1016/j.celrep.2015.12.011 26725119

[58] Plant EP, Ye Z. A codon-pair bias associated with network interactions in influenza A, B, and C genomes. Front Genet. 2021;12:699141. doi: 10.3389/fgene.2021.699141 34295355 PMC8290168

[59] Khedkar PH, Osterrieder N, Kunec D. Codon pair bias deoptimization of the major oncogene Meq of a very virulent Marek’s disease virus. J Gen Virol. 2018;99(12):1705–1716. doi: 10.1099/jgv.0.001136 30113295

[60] Wang L, Zhao H, Wang Z, et al. An evolutionary perspective of codon usage pattern, dinucleotide composition and codon pair bias in Prunus necrotic ringspot virus. Genes (Basel). 2023;14(9):1712. doi: 10.3390/genes14091712 37761852 PMC10530913

[61] Tulloch F, Atkinson NJ, Evans DJ, et al. Rna virus attenuation by codon pair deoptimisation is an artefact of increases in CpG/UpA dinucleotide frequencies. Elife. 2014;3:e04531. doi: 10.7554/eLife.04531 25490153 PMC4383024

[62] van Weringh A, Ragonnet-Cronin M, Pranckeviciene E, et al. HIV-1 modulates the tRNA pool to improve translation efficiency. Mol Biol Evol. 2011;28(6):1827–1834. doi: 10.1093/molbev/msr005 21216840 PMC3098512

[63] Wilusz JE. Controlling translation via modulation of tRNA levels. Wiley Interdiscip Rev RNA. 2015;6(4):453–470. doi: 10.1002/wrna.1287 25919480 PMC4478206

[64] Iroegbu CU, Pringle CR. Genetic interactions among viruses of the Bunyamwera complex. J Virol. 1981;37(1):383–394. doi: 10.1128/JVI.37.1.383-394.1981 7218427 PMC171016

[65] Barbosa NS, Concha JO, DaSilva LLP, et al. Oropouche virus glycoprotein topology and cellular requirements for glycoprotein secretion. J Virol. 2023;97(1):e0133122. doi: 10.1128/jvi.01331-22 36475765 PMC9888203

[66] Foster JE, López K, Eastwood G, et al. Phylogenetic characterization of orthobunyaviruses isolated from Trinidad shows evidence of natural reassortment. Virus Genes. 2023;59(3):473–478. doi: 10.1007/s11262-023-01973-5 36763228 PMC10199832

[67] Komar AA. The yin and yang of codon usage. Hum Mol Genet. 2016;25(R2):R77–R85. doi: 10.1093/hmg/ddw207 27354349 PMC6372012

[68] Bulmer M. Coevolution of codon usage and transfer RNA abundance. Nature. 1987;325(6106):728–730. doi: 10.1038/325728a0 2434856

