# Supplementary material for: Mapping evolutionary paradigm of Oropouche virus driven by dinucleotide bias and context-dependent codon bias
Source: Virulence. 2025 Dec 4;16(1):2600128. doi: 10.1080/21505594.2025.2600128 (PMC12688272; doi:10.1080/21505594.2025.2600128)
Supplement: Table S1 Information about OROV.doc [file KVIR_A_2600128_SM9548.doc]

Table S1 Oropouche virus strains selected in the study

| Strain | Host | Year of isolation | Genbank access number | | |
| --- | --- | --- | --- | --- | --- |
|  |  |  | S segment | M segment | L segment |
| BeAn 208402 | Bradypus tridactylus | 1971 | MG747542.1 | MG747543.1 | MG747544.1 |
| BeAn 208819 | Bradypus tridactylus | 1971 | MG747545.1 | MG747546.1 | MG747547.1 |
| BeAn 626990 | Callithrix | 2000 | MG747521.1 | MG747522.1 | MG747523.1 |
| BeAr 19886 | Ochlerotatus serratus | 1960 | MG747524.1 | MG747525.1 | MG747526.1 |
| BeAr 136921 | Culex quinquefasciatus | 1968 | MG747536.1 | MG747537.1 | MG747538.1 |
| BeAr 366927 | Culicoides paraensis | 1979 | MG747551.1 | MG747552.1 | MG747553.1 |
| BeH 29086 | Homo sapiens | 1961 | MG747527.1 | MG747528.1 | MG747529.1 |
| BeH 29090 | Homo sapiens | 1961 | MG747530.1 | MG747531.1 | MG747532.1 |
| BeH 121923 | Homo sapiens | 1967 | MG747533.1 | MG747534.1 | MG747535.1 |
| BeH 385591 | Homo sapiens | 1980 | MG747554.1 | MG747555.1 | MG747556.1 |
| BeH 390242 | Homo sapiens | 1980 | MG747509.1 | MG747510.1 | MG747511.1 |
| BeH 532314 | Homo sapiens | 1994 | MG747557.1 | MG747558.1 | MG747559.1 |
| BeH 532422 | Homo sapiens | 1994 | MG747560.1 | MG747561.1 | MG747562.1 |
| BeH 532490 | Homo sapiens | 1994 | MG747563.1 | MG747564.1 | MG747565.1 |
| BeH 532500 | Homo sapiens | 1994 | MG747566.1 | MG747567.1 | MG747568.1 |
| BeH 543629 | Homo sapiens | 1996 | MG747572.1 | MG747573.1 | MG747574.1 |
| BeH 543760 | Homo sapiens | 1996 | MG747575.1 | MG747576.1 | MG747577.1 |
| BeH 543857 | Homo sapiens | 1996 | MG747578.1 | MG747579.1 | MG747580.1 |
| BeH 708717 | Homo sapiens | 2006 | MG747599.1 | MG747600.1 | MG747601.1 |
| PPS 522 H 669314 | Homo sapiens | 2003 | MG747581.1 | MG747582.1 | MG747583.1 |
| PPS 523 H 669315 | Homo sapiens | 2003 | MG747584.1 | MG747585.1 | MG747586.1 |
| TRVL9760 | Homo sapiens | 1955 | KP026181.1 | KP026180.1 | KP026179.1 |
| TVP-19249 GML-444672 | Homo sapiens | 1989 | KP795077.1 | KP795076.1 | KP795075.1 |
| TVP-19254 GML-480914 | Homo sapiens | 1989 | KP795083.1 | KP795082.1 | KP795081.1 |
| 0200178W | Homo sapiens | 2020 | OP244879.1 | OP244878.1 | OP244877.1 |
| BeH 355173 | Homo sapiens | 1978 | MG747548.1 | MG747549.1 | MG747550.1 |
| BeH 541140 | Homo sapiens | 1994 | MG747569.1 | MG747570.1 | MG747571.1 |
| BeH 707287 | Homo sapiens | 2006 | MG747596.1 | MG747597.1 | MG747598.1 |
| BeH 708139 | Homo sapiens | 2006 | MG747593.1 | MG747594.1 | MG747595.1 |
| BeH505764 | Homo sapiens | 1991 | PP357050.1 | PP357049.1 | PP357048.1 |
| FPI21318 | Homo sapiens | 2024 | PP966966.1 | PP966974.1 | PP966982.1 |
| ILMD_TF29 | Homo sapiens | 2015 | PP154170.1 | PP154171.1 | PP154172.1 |
| LET-882 | Homo sapiens | 2021 | OP244885.1 | OP244884.1 | OP244883.1 |
| OROV/EC/Esmeraldas/155/2016 | Homo sapiens | 2016 | MK506819.1 | MK506824.1 | MK506829.1 |
| OV/Homo sapiens/Haiti-1/2014 | Homo sapiens | 2014 | MN264269.1 | MN264268.1 | MN264267.1 |
| PMOH 682426 | Homo sapiens | 2004 | MG747587.1 | MG747588.1 | MG747589.1 |
| PMOH 682431 | Homo sapiens | 2004 | MG747590.1 | MG747591.1 | MG747592.1 |
| TVP-19259/IQT-7085 | Homo sapiens | 1998 | KP795095.1 | KP795094.1 | KP795093.1 |
| TVP-19260/MD-203 | Homo sapiens | 1994 | KP795098.1 | KP795097.1 | KP795096.1 |
| BeAn 626990 | Homo sapiens | 2000 | AY117135.3 | MG747522.1 | MG747523.1 |
| BeH 472433 | Homo sapiens | 1988 | MG747512.1 | MG747513.1 | MG747514.1 |
| BeH 498913 | Homo sapiens | 1990 | MG747602.1 | MG747603.1 | MG747604.1 |
| BeH 543100 | Homo sapiens | 1996 | MG747503.1 | MG747504.1 | MG747505.1 |
| BeH 389865 | Homo sapiens | 1980 | MG747506.1 | MG747507.1 | MG747508.1 |
| BeH 472435 | Homo sapiens | 1988 | MG747515.1 | MG747516.1 | MG747517.1 |
